# Epidemiological and clinical aspects of immunoglobulin A vasculitis in childhood: a retrospective cohort study

**DOI:** 10.1186/s13052-021-01182-6

**Published:** 2021-12-15

**Authors:** Breda Luciana, Carbone Ilaria, Casciato Isabella, Cristina Gentile, Eleonora Agata Grasso, Giulia Di Donato, Chiarelli Francesco, Alberto Verrotti

**Affiliations:** 1 Department of Paediatrics, University of Chieti, Via dei Vestini 31, Chieti, Italy; 2 Department of Paediatrics, University of Perugia, Piazza dell’Università 1, Perugia, Italy

**Keywords:** Immunoglobulin A vasculitis, Henoch-Schӧnlein purpura, Pediatrics

## Abstract

**Background:**

A retrospective study was conducted in order to investigate and describe the characteristics of Immunoglobulin A vasculitis (IgAV), previously known as Henoch-Schӧnlein purpura, in the paediatric population of a community-based healthcare delivery system in the Italian region of Abruzzo.

**Methods:**

This is a population-based retrospective chart review of the diagnosis of IgAV in children ages 0 to 18, admitted to the Department of Paediatrics of Chieti and Pescara between 1 January 2000 and 31 December 2016. All children enrolled presented with clinical symptoms and laboratory findings and met the EULAR/PRINTO/PRES 2008 criteria.

**Results:**

Two-hundred-eight children met the criteria for IgAV, with the highest incidence reported among children below 7-years of age. A correlation with recent infections was found in 64% of the cohort; the onset was more frequently during the winter and fall. Purpura had a diffuse distribution in the majority of patients; joint impairment was the second most frequent symptom (43%), whereas the gastrointestinal tract was involved in 28% of patients.

**Conclusions:**

Hereby, we confirm the relative benignity of IgAV in a cohort of Italian children; with regards to renal involvement, we report a better outcome compared to other studies. However, despite the low rate of renal disease, we observed a wide use of corticosteroids, especially for the treatment of persistent purpura.

## Background

IgA Vasculitis (IgAV), previously known as Henoch–Schönlein Purpura (HSP), is a systemic vasculitis, characterized by polymorphonuclear leukocyte inflammatory infiltration of small blood vessels along with IgA1-predominant immune deposits [1].

IgAV represents the most common vasculitis in childhood, with a reported annual incidence rate of 3–27 cases per 100.000 [1]; indeed, IgAV is between 2 and 33 times more frequent in children than in adults, with approximately 90% of cases occurring between 2 and 10 years of age and a peak of incidence of between 4 and 7 years [2].

It is usually a self-limiting disease, with an average duration of 4 weeks; it may have a remitting-relapsing course, especially in the first 3 months after initial resolution. The range of clinical manifestations for IgAV is very broad. Palpable purpura is usually the first symptom of HSP; however, joint, gastrointestinal and renal involvement can occur [3]. Long-term complications are rare and include persistent hypertension and end-stage kidney disease. Rarely, when complicated, IgAV can be fatal [4].

The aim of this study is to describe the epidemiological, clinical, laboratory, and evolution characteristics of patients with IgAV in an Italian paediatric cohort.

## Methods

This is a population-based retrospective chart review of the diagnosis of IgAV in children ages 0 to 18, admitted to the Department of Paediatrics of Chieti and Pescara between 1 January 2000 and 31 December 2016. Chieti and Pescara are two provincial capitals of the central Italy region of Abruzzo, with a total area of 3830 km^2^. According to the Italian National Institute of Statistics Records the total population of the two provinces is estimated to be nearly 701.867 people, 17% of which are aged < 18 years. The population is mainly of Caucasian origin.

All children who met the criteria formulated by European Alliance of Associations for Rheumatology (EULAR)/ Paediatric Rheumatology INternational Trials Organisation (PRINTO)/ Paediatric Rheumatology European Society (PRES) in 2008 [5] were enrolled. According to these criteria, a child is classified as having IgAV if s/he has typical purpura (mandatory criterion) with lower limb predominance and one of the following: 1) abdominal pain; 2) typical histopathologic findings (leukocytoclastic vasculitis or proliferative glomerulonephritis with predominant IgA deposits), 3) arthralgia/arthritis; 4) renal involvement. Abdominal involvement was defined as the presence of abdominal pain, vomiting, gastrointestinal bleeding or intussusception. Renal involvement was defined according to the presence of any of the following findings: (1) proteinuria (> 0.3 g/24 h or > 30 mmol/mg of urine albumin/creatinine ratio on a spot morning sample); (2) hematuria or red blood cell casts (> 5 red blood cells/high power field or red blood cells casts in the urinary sediment or ≥ 2+ on dipstick). Renal biopsy was performed in the patients with nephrotic syndrome, nephrotic-range proteinuria, or persistent non-nephrotic proteinuria.

All children were examined upon admission and at subsequent follow-up visits.

All medical records of patients were analyzed to retrieve date and place of birth, age at disease presentation, sex, ethnicity, laboratory results, kidney involvement and follow-up, treatments, as well as any recurrence of the disease.

Furthermore, all patients were questioned for predisposing factors including infections, vaccine, etc. at the first visit.

To minimize entry error, all data were double checked. Continuous outcome measures were described as measures of central tendency and dispersion based on sample distribution. Microsoft Excel 2016 was used as a database.

The annual rate of IgAV hospitalization was estimated by dividing the number of IgAV hospitalizations (numerator) by the corresponding subgroup population (denominator). Hospitalization rate was expressed on a per 100.000 children basis.

## Results

Two hundred and eight patients met the EULAR/PRINTO/PRES criteria for IgAV; epidemiologic, demographic and clinical characteristics of the cohort are described in Table 1.

**Table 1:** Epidemiological data, etiologic factors and clinical features in 208 children with IgAV

Demographic characteristics of the cohort	***n*** = 208	% of the cohort
Mean age **(years)**, (Range)	6.44 (0–18)	
Males	108	51
Female	100	49
Ratio M:F	1.08:1	
**Seasonal pattern**		
Spring-summer	63	30
Autumn-winter	145	70
**Possible etiological factors**		
Total	132	63
Identified	59	28
Group A beta hemolytic streptococcus	40	19
Adenovirus	9	4
Mycoplasma	5	2
Coxsackievirus	2	1
Respiratory Syncytial Virus	2	1
Epstein Barr Virus	1	0.5
**Symptoms**		
Purpura	208	100
Diffuse	117	56
Leg and buttocks	23	11
Arthritis	48	23
Major joints	44	22
Minor joints	29	14
Abdominal pain	52	25
GI complications	7	4
Thickening of the bowel wall	3	2
Peritoneal effusion	2	1
Intussusception	2	1
Renal involvement	**58**	28
Proteinuria	41	20
Hematuria	38	18
Both	21	10
Mesangial IgA deposits	3	1
Scrotal involvement	7	4
Cardiovascular involvement	5	3
Mild ECG alterations	2	
Cardiac abnormalities	3	
Pulmonary complications	1	0.5

### Epidemiological and demographic characteristics

Between 2000 and 2016, the mean annual rate of hospitalization of patients diagnosed with IgAV was 12 patients (range 4–24), with the highest incidence reached in 2013 when 24 new cases were recorded.

The mean age at hospitalization was 6.44 years (median 5.76, range 0–18), with a similar male/female distribution (108 males and 100 females). Thirthy-seven children (77%) experienced the onset of the disease below the age of 7 years. IgAV occurred more frequently in winter and fall (*n* = 145, 70%) compared to summer and spring (*n* = 63, 30%) and followed an infection in 132 patients (63%); an infectious agent was identified in 59 patients (28%).

### Clinical manifestations

Purpura was present in all patients and was reported to be diffuse in 117 (56%); however, a small group (11%) presented cutaneous manifestation localized only in the lower extremities (gluteus and limbs). Arthralgia was complained by 89 patients (43%), whereas arthritis was observed in 48 children (23%) with lower limb large joints being the most affected.

Gastrointestinal symptoms were reported in 59 patients (28%), with abdominal pain being the most common (25%). Seven patients had serious complications: 3 presented thickening of the bowel walls, 2 manifested peritoneal effusions, 2 experienced intestinal intussusceptions and 1 complained of gastritis.

Renal involvement was detected in 59 children (28%), manifesting as proteinuria (20%) and hematuria (18%) within a range of 2 days to 2 weeks from the onset of symptoms. Renal biopsy was performed in 4 patients and IgA deposits were detected in 3 (1%).

Moreover, 7 patients experienced scrotal swelling, 5 had cardiac involvement and 1 pulmonary symptoms, with cough and pulmonary opacity at chest x-ray. With regard to cardiac involvement, 2 patients had mild ECG alterations (right bundle branch block and respiratory sinus arrhythmia), whereas in 3 patients subaortic interventricular defect, mild mitral insufficiency and thickness of mitral valve leaflets were observed after undergoing echocardiographic examinations.

### Laboratory tests

Leukocytosis (> 15.000) was observed in 50 patients (24%); thrombocytosis was observed in 14 patients (7%). Erythrocyte sedimentation rate (ESR) and C reactive protein (CRP) were elevated respectively in 99 (48%) and 98 (47%) patients. The median ESR was 27 mm/hour (r = 2–65) and the median CRP was 1.6 mg/dL (r = 0–19). Twenty-two patients (11%) had anemia, with a mean Hb of 10.9 mg/dl. C3 and C4 values were obtained in 174 patients (84%): in 133 patients (76%) C3 was within the normal range, whereas C3 was elevated in 40 patients (23%) and depressed in only 1 patient. C4 was normal in 138 patients (79%), elevated in 34 (20%) and depressed in 2 (1%). Leukocytes were detected in the urine of 75 patients (36%), while occult fecal blood was reported in 69 children (33%).

### Treatment

The majority of patients in the current series received drug therapy; the most commonly prescribed drugs were Non-Steroidal Anti-Inflammatory drugs (NSAIDs) (67% of the cohort).

Corticosteroids (CS) were administered in 62 patients (30% of the cohort) because of renal involvement (13%), persistent skin lesions (9%), severe abdominal pain (5%) or scrotal involvement (2%). The mean duration of steroid therapy was 1 month, and the median initial or equivalent dose was 1.5 mg/kg/day (r 0.75–2.0 mg/kg/day, SD 0.4).

Immunosuppressive therapy was required in 2 patients with renal involvement; the drugs chosen were azathioprine (AZA) and cyclophosphamide (CYC). Both children had initially received CS. The first patient was a 7-year-old child with persistent hematuria and proteinuria; azathioprine was administered with gradual improvement at a dosage of 1 mg/kg/day. The second patient was a 15-year-old girl with IgA nephropathy who had no response to CS; she underwent a renal biopsy and received cyclophosphamide at a dose of 2 mg/kg/day. Both patients showed a good response to immunosuppressive drugs and had a favorable outcome. No severe infections or adverse reactions were reported during treatment.

## Discussion

IgAV is one of the most common forms of vasculitis among children, most frequently occurring before the age of 10, characterized by polymorphonuclear leukocyte inflammatory infiltration of small blood vessels along with IgA1-predominant immune deposits [1]. Elevated serum galactose-deficient IgA1 levels are seen in IgAV, and abnormal IgA1 glycosylation is believed to be the main pathogenetic mechanism [1]. Although the etiology remains unknown, a possible link between genetic predisposition and environmental factors could play a role in the pathogenesis of IgAV [6]. Genome-wide association studies have demonstrated the influence of mutations on the predisposition to this condition and also on the way the disease manifests itself [1]. Recent studies support a strong association between IgAV and HLA; in the Caucasian population, an important pathogenic role has been demonstrated for HLA-DRB1*01 alleles, whereas HLA-DRB1*03 seems to be protective [7]. Furthermore, angiotensin-converting enzymes (ACE), Interleukin 18 (IL-18) and HLA-B*35 genes have been associated with a more aggressive renal phenotype [1].

In the present study, 208 children met IgAV criteria over a period of 17 years; 190 patients in our study (91%) were < 10 years of age at the time of diagnosis. This is in line with the results from an Italian retrospective study [8] and with data extracted from similar paediatric cohorts from the US and Asia [9–11].

In contrast to the majority of retrospective studies from other countries, which reported a greater male prevalence [8–12], a similar male/female distribution (M:F ratio = 1.08:1) was observed in our cohort. As previously reported, the occurrence of IgAV follows a seasonal variability with a fall-winter incidence peak [13], suggesting the role of climate-related environmental triggers, particularly for infections. Group A streptococcal infections have long been considered the exclusive triggering factor of this vasculitis. However, a wide variety of further viral, bacterial, and perhaps protozoan infectious agents may be associated to IgAV onset [6].

In our cohort 70% of patients developed IgAV in autumn-winter, as compared to 30% in spring-summer. A recent infection was found in 132 patients (64%), with group A beta-hemolytic streptococcus being the most common agent identified (20%). Many other trigger factors, such as vaccinations and drugs have been described, but further studies are needed to confirm their role due to the conflicting results obtained so far [13].

### Clinical features

We considered the main clinical features of IgAV and performed a comparative analysis between our patients and those from other retrospective studies from Italy and other regions of the world.

Palpable purpuric rash and subcutaneous edema are the cutaneous hallmarks of IgAV. Purpura has a typical acute onset, a symmetrical distribution, mainly on buttocks and lower extremities; it can be seen less frequently on the face and torso. Eruptions typically occur in crops, do not disappear with pressure and usually present a defined margin, with a size ranging from pinpoint to several centimeters [3].

Skin lesions characterized the onset of the disease in 90% of our cases, and were frequently associated with articular or abdominal symptoms. Purpura was reported in all patients, and was diffuse in 56% of them; however, in 11% patients, it was localized in the lower extremities (gluteus and limbs). No cases of bullous-hemorrhagic purpura were reported.

Musculoskeletal involvement represents the second most frequent manifestation of the disease (up to 70–90% of patients) and can manifest as arthralgia or arthritis and in 5–25% of patients may precede the onset of purpura [14]. Arthritis frequently has an oligo-articular pattern, with joints of the feet and ankles being the most commonly involved followed by knees, wrists, elbows, and hands [1].

Importantly, arthritis is usually self-limited and does not cause any residual abnormalities such as joint erosions. In our cohort, arthralgia affected 43% patients, being the most frequent manifestation. Arthritis was reported in 23% children. Similarly to the data reported in literature, in our series, major joints of lower limbs were the most frequently involved.

According to current literature, gastrointestinal (GI) manifestations are the third manifestation reported in order of frequency [15]. A higher frequency was only reported in Japanese children, making GI manifestations more frequent than musculoskeletal involvement [16]. In the present study, GI symptoms were reported in 28% of patients, with abdominal pain being the most common. Other frequent symptoms were GI bleeding (9%), and diarrhoea (2.5%) [16]. Serious complications were diagnosed in 7 patients; no cases of GI bleeding were diagnosed. Other uncommon complications, such as malabsorption and exudative enteropathy did not occur in our cohort. Interestingly, a recent study has highlighted a possible correlation between GI symptoms and *Clostridium difficile* infection [17]; however, in our cohort we did not find any case of Clostridium infection.

Incidence of renal involvement ranges from 30 to 50% [1, 18] and has a key role in determining IgAV long-term prognosis, including mortality and morbidity. Clinical manifestations may vary from microscopic haematuria and/or proteinuria to nephritis, characterized by the deposition of extrarenal-IgA, C3 and other complement factors in the mesangium, subepithelial and subendothelial space, leading to an increased risk of chronic kidney disease. The proportion of the patients progressing to renal failure or end-stage renal disease varies from 1 to 7% [18]. Risk factors for nephropathy in the course of IgAV include male gender, being over the age of 10, the presence of severe gastrointestinal involvement, persistent purpura, relapses, arthritis/arthralgia, and laboratory abnormalities (leukocytosis above 15 × 109 /L, thrombocytosis above 500 × 109/L, elevated serum ant streptolysin O titer, and decreased serum c3 of the complement concentration) [19]. Renal involvement was diagnosed in 28% children of the cohort: proteinuria occurred in 20% and haematuria in about 18%, with 10% experiencing both conditions. No cases of end-stage renal failure or chronic renal insufficiency were reported; thus, we report a milder renal involvement compared to previous reports [8–11].

IgAV may also affect the reproductive system. Whereas only one case of female reproductive system involvement has been described [20], male genitalia is more frequently affected, with an incidence ranging between 2 and 38%. Edema and pain of the scrotum, spermatic cord and testis, epididymitis, orchitis, hematoma around the testis and testicular torsion are the most common manifestations [21]. In our cohort, scrotal swelling was found in only 3% of patients, a lower percentage compared to the ones reported by a previous Italian population-based study [8].

Although rare, cardiovascular involvement is possible, and myocarditis is the most common complication, though valvulitis and thromboses may also occur [22]. Indeed, in our cohort only 5 patients have experienced cardiac involvement; among these, 2 patients manifested mild ECG alterations (right bundle branch block and respiratory sinus arrhythmia), whereas in 3 patients subaortic interventricular defect, mild mitral insufficiency and thickness of mitral valve leaflets were observed after undergoing echocardiographic examinations. However, it was not possible to establish a clear correlation between these cardiac changes and IgAV, despite the execution of a further echocardiography 1 month after the onset of the purpura revealed a complete resolution of the picture in the last patient. No episodes of severe valvulitis or thrombosis have been recorded in our cohort. Interestingly, a recent retrospective study has shown that IgAV can be also associated with an increased risk of hypertension and chronic kidney disease [23].

Finally, sub-clinical lung impairment without respiratory symptoms has been frequently reported in literature, and severe lung complications such as diffuse alveolar haemorrhage can rarely occur [24]. In our cohort, only 1 patient showed respiratory symptoms due to pneumonia, with cough and mild reduced transparency on chest x-ray.

### Laboratory tests

IgAV diagnosis is clinical. Laboratory tests can be useful to exclude other diseases and identify complications, especially renal involvement. In everyday clinical practice, the routine laboratory testing of children with new-onset IgAV differs significantly among hospitals, though useful baseline studies often include renal function tests (such as urinalysis and the determination of urea, creatinine, and electrolytes in blood), complete blood count, coagulation profile, and ESR. Detecting immunoglobulin G antinuclear (ANA) or antineutrophil cytoplasmic auto-antibodies (ANCA) may help in ruling out other vasculitides. Skin biopsy is required in limited cases [25].

Recently, the role of other specific biomarkers has been assessed; among them, serum amyloid A was found to be the most valuable for the laboratory diagnosis of patients with IgAV [26].

In our study the most frequent findings were increased ESR, elevated CRP leucocytosis and anaemia; thrombocytosis was detected in 6% of patients. In contrast, a lower percentage of leukocytosis were reported in the South American paediatric population [9]. Faecal occult blood tests were positive in 69 children (33%).

Despite the key role played by complement system in IgAV pathogenesis, serum levels of C3 and C4 are within normal range for most patients, despite reductions of C3 and C4 levels can occur as a result of complement components consumption [27]. In our series serum C3 and C4 levels were measured in 139 children, and a reduction was demonstrated in only 3 patients, a lower percentage compared to what previously reported [8].

### Treatment

IgAV is often a self-limiting disease and supportive measures such as bed rest, adequate hydration, and monitoring of vital signs is enough in most cases [1].

Adequate analgesia with NSAIDs should be prescribed for IgAV associated arthropathy, if renal function is normal, despite the presence of microscopic haematuria [25].

CS should be considered in patients with nephritis, orchitis, cerebral vasculitis, pulmonary haemorrhage and severe GI involvement [1, 25, 28]. Purpura is not an indication for CS administration, with the exception of bullous-hemorrhagic rash in which, despite the lack of consensus, it may lead to a clinical improvement in these patients [29].

Specific treatment indications for IgAV nephritis are reported in the European consensus-based recommendations [25]. CS are considered the first line treatment both in mild and moderate nephritis, whereas immunosuppressive agents, including AZA, mycophenolate mofetil (MMF), or CYC, may be used as first- or second-line treatment according to the histopathological findings of renal biopsy. In contrast, severe IgAV nephritis immediately required high-dose CS and intravenous CYC to induce remission, and lower doses of CS combined with AZA or MMF as maintenance treatment [25].

Finally, the use of ACE inhibitors or angiotensin receptor blockers should be considered to prevent and/or limit secondary glomerular injury in children with persistent proteinuria lasting more than 3 months [30]. Other lines of therapy administered in severe or refractory IgAV patients include intravenous immunoglobulins, plasma exchange, colchicine and rituximab [31–34]. The therapeutic approach is summarized in Table 2 [insert Table 2].

**Table 2:** Drugs administered in the cohort studied

Drug	N. of patients (% of the whole cohort)	Treatment indications	n
NSAIDs	139 (67)	Joint involvement	58
		Abdominal pain	11
		Both	22
		Previous infection	21
		Other	27
Corticosteroids	62 (30)	Purpura	19
		Abdominal pain	10
		GI complications	5
		Scrotal involvment	5
		Renal involvement	27
Immunosuppressive drugs	2 (1)	IgA nephritis	1
		Persistent proteinuria	1

Sixty-seven percent of patients received NSAIDs, whereas CS were administered in 62 patients because of renal dysfunction (13% of the whole cohort), persistent skin lesions (9%), severe abdominal pain (5%) or scrotal involvement (2%). Immunosuppressive therapy was required in only 2 patients because of persistent proteinuria in the first case, and non-response to CS therapy in the second.

### Study limitations

There were several limitations in this study. The population only included hospitalized patients; hence, the ascertainment of patients may have been biased toward more severe phenotypes. Moreover, since data were collected retrospectively from the electronic health record database some children were lost at follow-up after the discharge from the Paediatric Department, with no information available about the occurring of relapses of the cohort studied.

## Conclusions

In conclusion, as already highlighted in a previous Italian study [8], we confirm the relatively mild course of IgAV in our cohort of Italian children, and report a positive outcome compared to other countries [9, 12]. Renal involvement was mild and showed a good response to first-line therapy in most cases. However, in our cohort, despite the low rate of renal impairment, we noticed a wide use of CS, especially in cases of persistent skin purpura.

## Data Availability

The datasets used and/or analysed during the current study are available from the corresponding author on reasonable request.

## Abbreviations

IgA: Immunoglobulin A
IgAV: Immunoglobulin A Vasculitis
HSP: Henoch–Schönlein Purpura
ACE: angiotensin-converting enzymes
IL-18: interleukin 18
ESR: erythrocyte sedimentation rate
CRP: c reactive protein
NSAIDs: non-steroideal anti-inflammatory drugs
CS: corticosteroids
AZA: azathioprine
CYC: cyclophosphamide
GI: gastrointestinal
ANA: antinuclear antibodies
ANCA: antineutrophil cytoplasmic antibodies
MMF: mycophenolate mofetil
