# Correction: Epidemiological and clinical aspects of immunoglobulin A vasculitis in childhood: a retrospective cohort study

**DOI:** 10.1186/s13052-022-01265-y

**Published:** 2022-05-19

**Authors:** Luciana Breda, Ilaria Carbone, Isabella Casciato, Cristina Gentile, Eleonora Agata Grasso, Giulia Di Donato, Francesco Chiarelli, Alberto Verrotti

**Affiliations:** 1 Department of Paediatrics, University of Chieti, Via dei Vestini 31, Chieti, Italy; 2 Department of Paediatrics, University of Perugia, Piazza dell’Università 1, Perugia, Italy

**Correction to: Ital J Pediatr 47, 1-7 (2021)**

**https://doi.org/10.1186/s13052-021-01182-6**

The original article [1] erroneously inverted several co-authors’ names; this has since been corrected.
